# Enhanced graph coevolution network for social network analysis using assimilation modified emotional algorithm

**DOI:** 10.1038/s41598-025-18482-0

**Published:** 2026-03-02

**Authors:** Hsiao-Hui Li, Po-Chun Chang, Yuan-Hsun Liao

**Affiliations:** 1 Department of Maritime Information and Technology, National Kaohsiung University of Science and Technology, Cijin Campus, Gaoxiong, 805301 Taiwan; 2 Department of Computer Science, Tunghai University, Taichung, 407224 Taiwan

**Keywords:** Graph machine learning, Social network analysis, Label propagation algorithm, Computational science, Information technology

## Abstract

This paper presents the Assimilation Modified Emotional (AME) algorithm, which is an enhanced version of the traditional label propagation algorithm (LPA) designed to address key challenges in social network analysis and emotional feature extraction. Traditional LPA methods, such as asynchronous label propagation and the Louvain algorithm, do not incorporate emotional representations and are often limited by local structural dependencies. The AME algorithm addresses these limitations by applying spectral algorithms, Markov chains, graph coarsening, and link prediction to simulate and optimize emotional transitions within the network. In addition, the AME algorithm enhances label representation through multi-label encoding, which allows for more accurate simulation of dynamic emotional states. Experimental results show that the AME algorithm achieves better performance than traditional LPA methods in terms of both accuracy and loss values. These findings indicate that the AME algorithm has strong potential for improving AI models used in social network analysis and emotional feature extraction.

## Introduction

The label propagation algorithm (LPA)^1–3^, a powerful semi-supervised learning method for inferring label features, plays a significant role in social network analysis^4^. Moreover, LPA effectively manages the propagation of neighbor features as community flows^5^, making it a recommended approach to enhance model performance. Label propagation algorithms represent a distinct subset of community detection methods. Common community detection approaches include the asynchronous label propagation algorithm^6^, the Louvain algorithm^7^, the fast label propagation algorithm^8^, and the greedy modularity algorithm^9^. Among these, the asynchronous label propagation algorithm^6^ and the fast label propagation algorithm^8^ are particularly prominent representatives of label propagation techniques.

Traditional label propagation algorithms encounter two primary challenges: excessive dependence on local structural information and insufficient consideration of the origin of label determination. Asynchronous label propagation^6^ and the Louvain algorithm^7^ face significant limitations in real-world applications. Traditional LPA may be limited to local structural configurations^10^, leading to suboptimal partitions in certain cases. Traditional LPA focuses solely on labeling, encoding, and neighborhood structure, overlooking the semantic representation of label types^11^.

To overcome these limitations, we propose a novel algorithm, the Assimilation Modified Emotional (AME) algorithm, which incorporates emotional semantics and global information dynamics into the label propagation process. The motivation of the AME algorithm is to reduce overreliance on local structural information and address the issue of label encoding. The encoding technique typically involves selecting the label with the highest probability from the output of a deep learning model’s flattened layer^12–14^. For example, the model was trained with three label classes: “Happy”, “Fear”, and “Sad”. After training, two test samples are fed into the model, producing two label probability distributions: {“Happy”: 0.43, “Fear”: 0.33, “Sad”: 0.24} and {“Happy”: 0.93, “Fear”: 0.05, “Sad”: 0.02}. After one-hot encoding, both probability arrays result in the same final predicted label: “Happy.” However, the underlying emotional meanings of the two predictions are clearly different. Despite this, the one-hot encoding process represents both inputs identically in the final output. To address the issue of overreliance on local structural information, the AME algorithm simulates the assimilation effect^15–18^ and implements a Markov chain to model emotional interaction probabilities^19–21^. Moreover, the AME algorithm applies a graph coarsening algorithm^22^ to reduce information propagation within communities. To improve the handling of label encoding, the AME algorithm adopts multi-label encoding instead of single-label encoding, allowing agents to represent multiple emotions. It employs a Markov chain process for dynamic emotional transitions, ensuring more realistic simulations.

The experimental results clearly demonstrate the effectiveness of the AME algorithm. In the Methods section, we introduced validation experiments for the core components: the Multiple Probability Interactive Markov Chain and the Markov Chain-based implementation of Graph Coarsening. The results confirmed the validity of these core components. In the Experiments and Discussion section, graphs were generated based on the Erdős–Rényi^23,24^ and Barabási–Albert^25,26^ models. Using this approach, two real-world text datasets, the Kaggle Emotion Detection dataset^27^ and the CARER dataset^28^, were annotated with emotion labels using the Emotion English DistilRoBERTa-base transformer^29^. Six graphs were generated for each model. The emotional labels from the annotated datasets were used as the initial emotional weights for each graph. This step was necessary because the graphs, including the Erdős–Rényi Graph, Barabási–Albert Graph, Facebook combination real-world graph dataset^30^, and email-Eu-core real-world dataset^31,32^, did not contain any inherent emotional probabilities. The performance of five algorithms was evaluated, including the Assimilation Modified Emotional algorithm, the asynchronous label propagation algorithm, the Louvain algorithm, the fast label propagation algorithm^8^, and the greedy modularity algorithm^9^. While test accuracy is emphasized as the primary comparison metric in the introduction, it is also worth noting that the AME algorithm consistently outperformed all other algorithms in terms of both validation accuracy and loss values across all three experiments and evaluated graph types. This consistent and comprehensive performance further demonstrates the robustness and effectiveness of the proposed method.

In both Experiment 1 and Experiment 2, the AME algorithm consistently outperformed the other methods. These experiments respectively utilized the Erdős–Rényi^23,24^ and Barabási–Albert^25,26^ models to generate graphs, with each model producing six graphs. A total of five algorithms were evaluated. For the first three graphs, the initial emotional probabilities were derived from the Emotion English DistilRoBERTa-base transformer^29^, applied to the Kaggle Emotion Detection dataset ^27^. For the latter three graphs, the same transformer^29^ was applied to the CARER dataset^28^. In Experiment 1, the AME algorithm achieved test accuracies of 98.8, 100, 100, 97.5, 100, and 98.8%, all of which were the highest among the five evaluated algorithms. Similarly, in Experiment 2, the AME algorithm attained test accuracies of 100, 98.3, 99.4, 97.5, 99.2, and 100%, again outperforming all other methods. In Experiment 3, two real-world networks, the Facebook social circles dataset^30^ and the email-Eu-core network^31,32^, were each evaluated twice using emotional labels derived from the Kaggle Emotion Detection dataset^27^ and the CARER dataset^28^, respectively. The AME algorithm once again achieved the best overall performance, with test accuracies of 99, 99.6, 99.6, and 100%, respectively. Compared with five baseline community detection algorithms, the Assimilation Modified Emotional algorithm, the Asynchronous Label Propagation Algorithm^6^, the Louvain algorithm^7^, the Fast Label Propagation Algorithm^8^, and the Greedy Modularity Optimization Algorithm^9^, the AME algorithm achieved the best results in terms of test accuracy, validation accuracy, and loss value. Based on these experimental findings, it can be conclusively affirmed that the Assimilation Modified Emotional algorithm outperforms the other methods and is the most effective among them.

In summary, this paper introduces the AME algorithm as an enhanced approach to label propagation in social network analysis. Unlike traditional methods, AME addresses two main challenges: the overreliance on local structural information and the limitations of single-label encoding. By employing a Markov chain^19–21^ to model emotional probability transitions and applying graph coarsening^22^ to simplify network structures, AME provides a more accurate and realistic labeling process. Experimental results on both synthetic and real-world datasets demonstrate that AME consistently outperforms existing algorithms, validating the effectiveness of its core design.

## Methods

This section outlines the objective function, algorithmic process, implementation details, and validation experiments. The validation experiment compares the performance of the algorithm with and without integrating the core design components.

### Objective function

The objective function of the AME algorithm is to enhance community-level feature representation for neural network model learning^33^ and the generalization across different graph structures. Traditional label propagation algorithms face two key issues: an overreliance on local structural information and a lack of consideration for label origin, particularly the use of one-hot encoding. To address these limitations, this study proposes an algorithm inspired by the assimilation effect^15–18^. The proposed algorithm retains the probability distribution of all possible labels and employs a Markov chain to model label interactions. This approach significantly strengthens the application of the assimilation effect in social network analysis. Additionally, the algorithm incorporates a graph coarsening technique to refine information propagation within sub-communities, thereby improving both generalization and model learning capacity of social network analysis.

### Proposed algorithm

The Assimilation Modified Emotional (AME) algorithm consists of four critical steps.

*Step 1* Emotion Prediction using a transformer model.

**Fig. 1:**
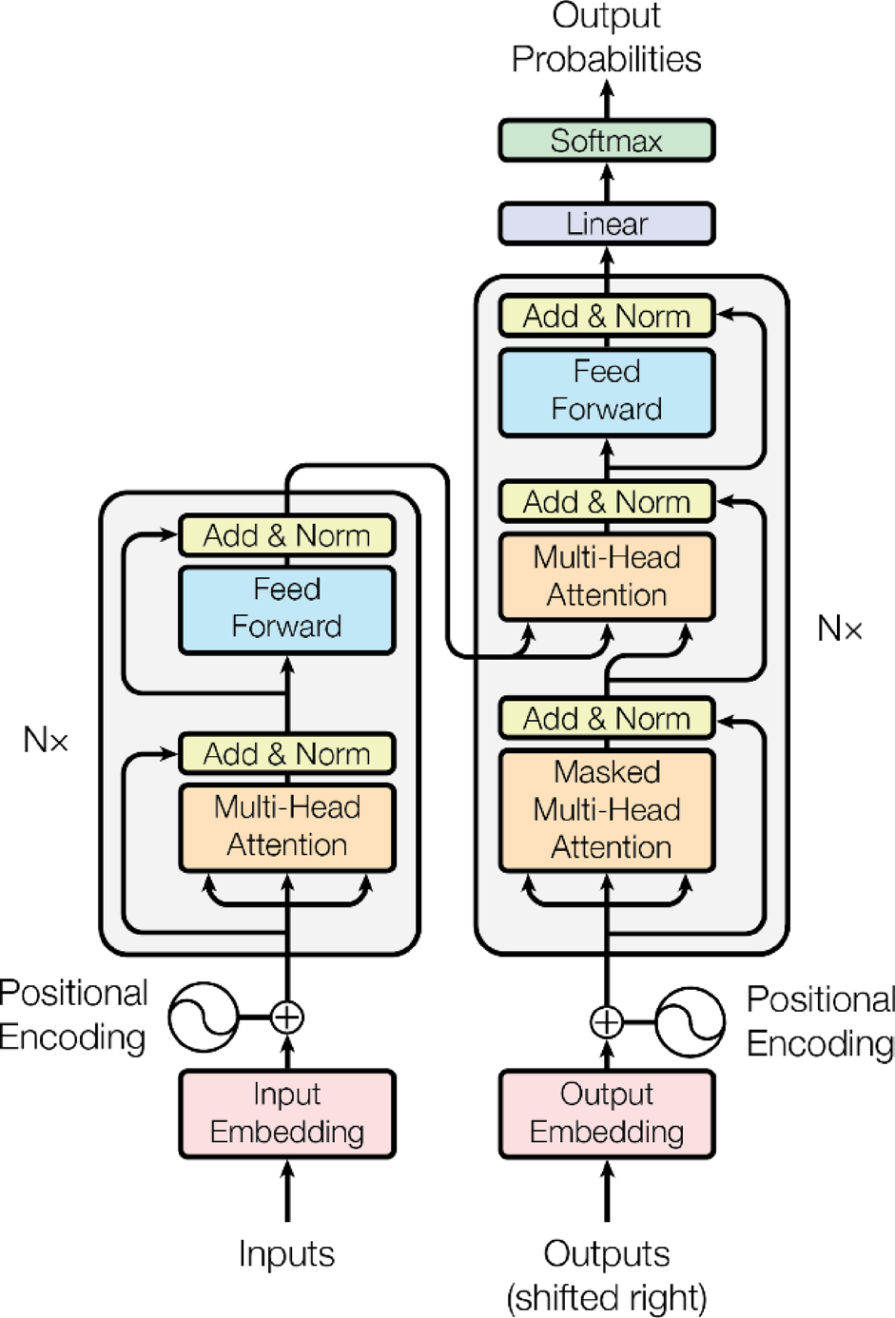
Transformer structure, resource from attention is all you need^39^.

The first step is to use a deep learning model^34,35^ to predict label probabilities and map each label probability to each node as weighted values. Deep learning models play a central role in processing emotion recognition tasks from text. AME and other related approaches commonly use convolutional neural networks (CNNs) to detect emotions from social media texts, such as tweets. These studies widely apply deep learning models to perform emotion recognition tasks. Deep learning models come in multiple forms, such as CNNs, long short-term memory networks (LSTMs), and recursive neural networks (RNNs). This study applies a fine-tuned transformer model to handle text-based emotion recognition tasks. The model employed is the Emotion English DistilRoBERTa-base^29^. Several studies have employed the Emotion English DistilRoBERTa-base model for emotion recognition tasks across various domains. For example, Rozado et al. (2022) utilized this model to conduct large-scale sentiment and emotion classification on news media headlines over time, aiming to analyze longitudinal trends in media tone^36^. Similarly, Kuang et al. (2022) applied the model in the context of music-to-text generation, using it to annotate the emotional content of musical pieces and improve descriptive text generation^37^. In the medical and health domain, Butt et al. (2022) leveraged the model to explore psycholinguistic patterns in social media posts, particularly focusing on emotional differences between rumour and non-rumour tweets related to health crises^38^. In summary, the Emotion English DistilRoBERTa-base model ^29^, developed from the transformer architecture^39^ and illustrated in Fig. 1, plays a key role as the default model for text-based emotion recognition in the AME algorithm.

*Step 2* Community Division via spectral clustering.

The second step is to use a spectral algorithm^40,41^ to divide the social network into multiple sub-communities and to identify the five nodes with the highest degree values^42^ as the core representative leaders of each sub-community. The spectral algorithm is a powerful community detection method widely applied in graph theory. Andrea Montanari and Nike Sun used the spectral algorithm for tensor completion tasks^43^. Florent Krzakala et al. applied the spectral algorithm to the community detection problem and proposed using the non-backtracking matrix as the basis for eigenvalue analysis ^44^. In conclusion, the spectral algorithm is a classic technique for community detection and is commonly used to split social networks into multiple sub-communities.

*Step 3* Emotional Interaction Modeling using a five-part Markov chain mechanism.

**Fig. 2:**
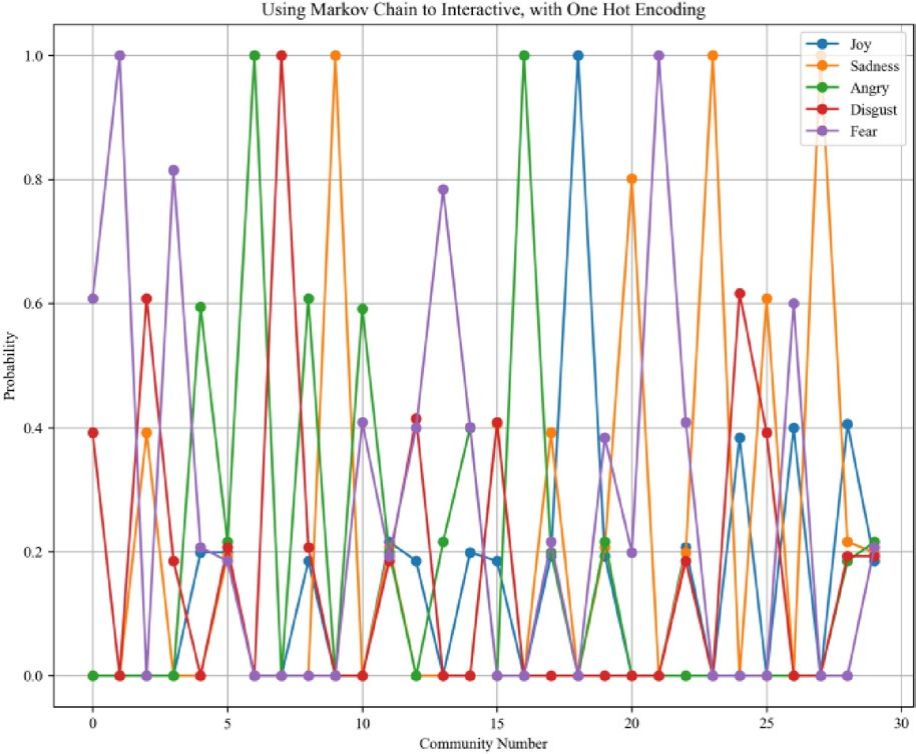
One-hot encoding process using the Markov chain.

**Fig. 3:**
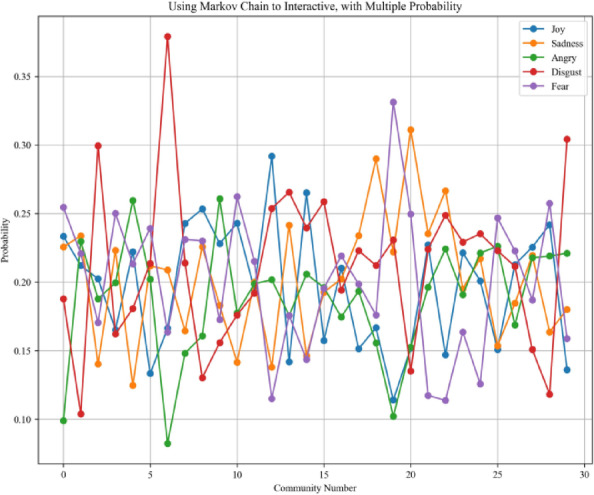
Multiple-probability encoding process using the Markov chain. This design is validated as a more effective design and is adopted in the algorithm proposed in this paper.

The third step is to loop through each community and use a Markov chain to adjust the weights of nodes within each sub-community. The Markov chain is a powerful mathematical framework used to model state transitions based on probabilistic rules. The Markov chain underlies many state-of-the-art techniques in probabilistic modeling. Gianfranco Bilardi et al. used Markov chains as input models to derive closed-form spectral expressions of digital signals generated through memoryless functions, enabling analysis of their frequency characteristics in digital systems^45^. Gibson et al. used Markov chain Monte Carlo (MCMC) methods to estimate parameters in stochastic epidemic models from incomplete observation data by simulating the underlying Markov process^46^. Therefore, the Markov chain underpins numerous theoretical models in probabilistic interaction modeling. In this step, the AME algorithm uses a Markov chain to update the weights of each role within its sub-community. The core design of this step lies in simulating the assimilation effect during the interaction process. The interactive Markov chain process used for encoding in the validation experiment is visualized in Figs. 2 and 3. The interaction process mainly pushes each role through emotional transitions to enhance the accuracy of community role assignments.

The Markov chain plays an important role in simulating emotional diffusion, and its implementation involves more sophisticated mathematical formulations. Therefore, we describe the design of the Markov chain in a more rigorous manner using detailed definitions. Specifically, the emotional interaction mechanism is structured as a five-part Markov chain process, which includes: (a) defining the emotional state space, (b) selecting leader nodes within each community, (c) assigning initial emotional states, (d) simulating emotional state transitions, and (e) estimating community-level emotional trends.

- Emotional state space definition:
  The Markov chain interaction process defines the state space of emotions. Emotional states are modeled as a finite and discrete set. Each individual occupies exactly one of these states at any given time step, and their emotional tendency is represented as a probability distribution vector over the elements of $\mathcal{S}$. The finite and discrete set of emotional states is denoted in Eq. (1).
  1 $$\mathcal{S}=\{{s}_{1},{s}_{2},\dots ,{s}_{n}\},n=|\mathcal{S}|$$
- Leader node selection:
  The Markov chain interaction process selects the top five most influential nodes from each community, which are identified as local leaders based on the spectral community detection results from Step 2. This set of representative nodes, denoted as the top t nodes of community $j$, serves as the foundation for constructing the community-level emotional transition matrix, as illustrated in Eq. (2).
  2 $$tranM{a}_{j}:=\{{V}_{sONet,i}{\}}_{i=1}^{5}$$
- Initial state assignment:
  This step of the Markov chain interaction process involves the initialization of emotional states and the construction of the transition matrix. Each node $z\in tranMaj$ is assigned an initial emotional distribution. This emotional distribution is derived from step 1 and calculated using the Emotion-English-DistilRoBERTa-base model^29^. It estimates the emotional label probabilities from real-world text datasets and serves as the initial emotional distribution for the social Markov chain interaction process. The initial emotional distribution is denoted by $\pi ,$ and is stored as a dictionary data structure, which logs the estimated emotional label probabilities for the corresponding text. The initial emotional states are denoted in Eq. (3).
  3 $${P}_{0}^{(z)}=\left[\begin{array}{cccc}{\pi }_{z1}^{\left(0\right)}& {\pi }_{z2}^{\left(0\right)}& \cdots & {\pi }_{zn}^{\left(0\right)}\end{array}\right], \sum_{j=1}^{n}{\pi }_{zj}^{(0)}=1$$
  The emotional state transitions follow the Markov property, where the next state depends only on the current state. This transition process is formally defined in Eq. (4).
  4 $$P=\left[\begin{array}{cccc}{p}_{11}& {p}_{12}& \cdots & {p}_{1n}\\ {p}_{21}& {p}_{22}& \cdots & {p}_{2n}\\ \vdots & \vdots & \ddots & \vdots \\ {p}_{n1}& {p}_{n2}& \cdots & {p}_{nn}\end{array}\right],\sum_{j=1}^{n}{p}_{ij}=1,\forall i$$
  For each community $Cj$, the Markov chain interaction process constructs a community-level transition matrix $tranMAj$ by averaging the transition matrices of its five representative nodes, as denoted in Eq. (5). The matrix serves as an aggregated model of emotional influence and diffusion behavior within community $j$.
  5 $$tranM{A}_{j}:=\frac{1}{5}\sum_{i=1}^{5}{P}_{transition}^{(i)}$$
- Transition simulation:
  The community-level emotional trend is estimated by averaging the predicted emotional distributions of the representative nodes. This step uses the Markov chain model to predict the emotional distribution of each representative node $z$ at time step $t$. The expression computes the emotional distribution of node $z$ in community $j$ after $t$ transitions, governed by the community-level matrix $tranMAj$. This process is denoted in Eq. (6).
  6 $$predDis{t}_{j}^{(z)}:={P}_{0}^{(z)}\cdot (tranM{A}_{j}{)}^{t}$$
- Community-level trend estimation:
  Community-level emotional trends are estimated by averaging the predicted emotional distributions across the representative nodes within each community. To extract group-level emotional tendencies, this step computes the mean of the predicted emotional distributions across the five representative nodes in community $j$. The resulting vector represents the average emotional distribution of the community at a given time step and is denoted in Eq. (7). The final long-term emotional distribution, also known as the asymptotic distribution, can be interpreted as a simulation of the assimilation effect once the emotional states stabilize.
  7 $$meanPre{d}_{j}:=\frac{1}{5}\sum_{i=1}^{5}predDis{t}_{j}^{(i)}$$

After the Markov chain interaction is completed, the AME algorithm proceeds to apply the graph coarsening algorithm^22^ to reduce the graph and extract a more concise and well-defined community structure. Notably, the application of the Markov chain^19–21^ results in uniform weighting across all nodes within each community, which enhances the effectiveness of the subsequent coarsening step. This third step of the AME algorithm is executed iteratively until all communities are fully processed. Graph coarsening is a powerful technique with various practical applications. For example, Wasim Sadiq and Maria E. Orlowska used graph reduction rules to simplify workflow models in order to detect structural conflicts like deadlocks and synchronization issues, and to analyze the correctness of the model^47^. Similarly, Wasim Sadiq and Maria E. Orlowska applied graph reduction techniques to control flow specifications of process models to identify structural conflicts that might hinder correct execution^48^. Based on these insights, this paper implements a graph coarsening algorithm as a key process.

*Step 4* Link Prediction and Final Output Construction

Step four uses the link prediction algorithm^49^ to connect each sub-community. The core process involves using node degree as the node feature, applying cosine similarity to measure the similarity between nodes, and finally predicting cross-graph links based on this similarity. Link prediction algorithms play an important role in social network analysis. For example, Huan Wang et al. used link prediction methods from the perspective of individual nodes to analyze edge generation by measuring how well each edge is explained by different algorithms, using the edge generation coefficient. The method constructs a diverse identification vector (Div), which represents the distribution of edges explained by each algorithm. Cosine similarity is then applied to compare this actual distribution with the expected distribution under a uniform node evolution assumption (EDiv), thereby quantifying each node’s evolution diversity^50^. Fulan Qian et al. proposed a link prediction algorithm (TPSR) that integrates strong ties within three degrees in social networks with the topological properties of nodes to improve the accuracy of predicting potential links between previously unconnected nodes^51^. In summary, the link prediction algorithm is widely applied across different studies. This paper implements the link prediction algorithm to connect each sub-community. Upon completion of the four computational steps, the algorithm yields a refined, efficient, and assimilation-effect-enhanced set of communities, which constitutes the final output.

**Algorithm 1:**
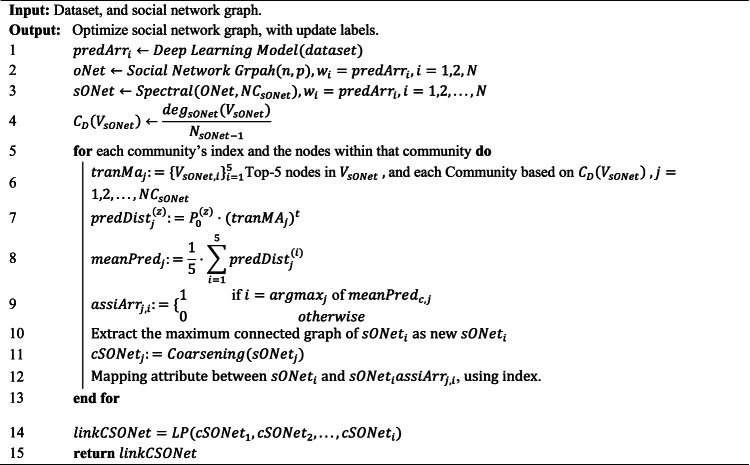
Assimilation modified emotional algorithm.

### Validation experiment for encoding design

The objective of this experiment is to evaluate whether retaining multiple probability values in the design of the AME algorithm yields better performance compared to the commonly used one-hot encoding approach. Four loss metrics are employed in this evaluation: Average Loss^52^, Average MAE^53^, Average RMSE^54^, and Average Huber Loss^55^. Ideally, lower loss values, approaching zero, indicate better model performance. According to the definitions of these loss functions, a value close to zero indicates that the predicted values closely align with the actual values, whereas a value approaching one suggests substantial deviation. In this experiment, emotion probabilities for each node are computed using a Markov chain-based method under two different encoding strategies: multi-probability and one-hot encoding. The resulting outputs are evaluated using the aforementioned loss metrics to determine the effectiveness of each encoding approach.

As shown in Table 1, the bolded values indicate the lower loss values in each corresponding column. It is evident from the table that the use of the multiple probability approach consistently yields lower loss values compared to the one-hot encoding method. This validation experiment on encoding design thus confirms the greater effectiveness of the multiple probability approach.

**Table 1:** The average loss, average MAE, average RMSE, and average Huber loss of Markov chain evaluation use multiple probabilities and one-hot encoding separately.

Label	Multiple probability					One hot encoding				
	Joy	Sadness	Angry	Disgust	Fear	Joy	Sadness	Angry	Disgust	Fear
Average loss^41^	**0.000947**	**0.000907**	**0.000961**	**0.000976**	**0.000926**	0.002678	0.002808	0.002955	0.002862	0.002658
Average MAE^42^	**0.1005**	**0.0955**	**0.1017**	**0.1023**	**0.0973**	0.2872	0.3022	0.3245	0.3029	0.2819
Average RMSE^43^	**0.1231**	**0.1159**	**0.1239**	**0.1266**	**0.1197**	0.4185	0.4183	0.4545	0.4258	0.4069
Average Huber loss^44^	**7.23e−05**	**6.60e−05**	**7.61e−05**	**8.09e−05**	**7.05e−05**	9.23e−04	9.13e−04	9.75e−04	9.33e−04	9.26e−04

The bolded value stands for the smaller loss value in the respective columns.

### Validation experiment for the graph coarsening design

The objective of this validation experiment is to demonstrate the effectiveness of the graph coarsening algorithm within the AME framework. The evaluation metrics include Precision^56^, Recall^57^, F1 score^58^, Accuracy^59^, Macro Average, and Weighted Average^60^, all of which are standard measures commonly used in classification tasks. In an ideal scenario, indicator values approaching 1 denote optimal performance, whereas values approaching 0 indicate poor performance. This validation experiment applies the AME algorithm for label propagation on an initial Erdős–Rényi graph under two configurations: one with graph coarsening^22^ and one without. The resulting community structures are subsequently used to train a Graph Convolutional Network (GCN), and the training outcomes are evaluated using the aforementioned evaluation metrics. This experimental design aims to assess whether incorporating graph coarsening enhances model performance.

**Fig. 4:**
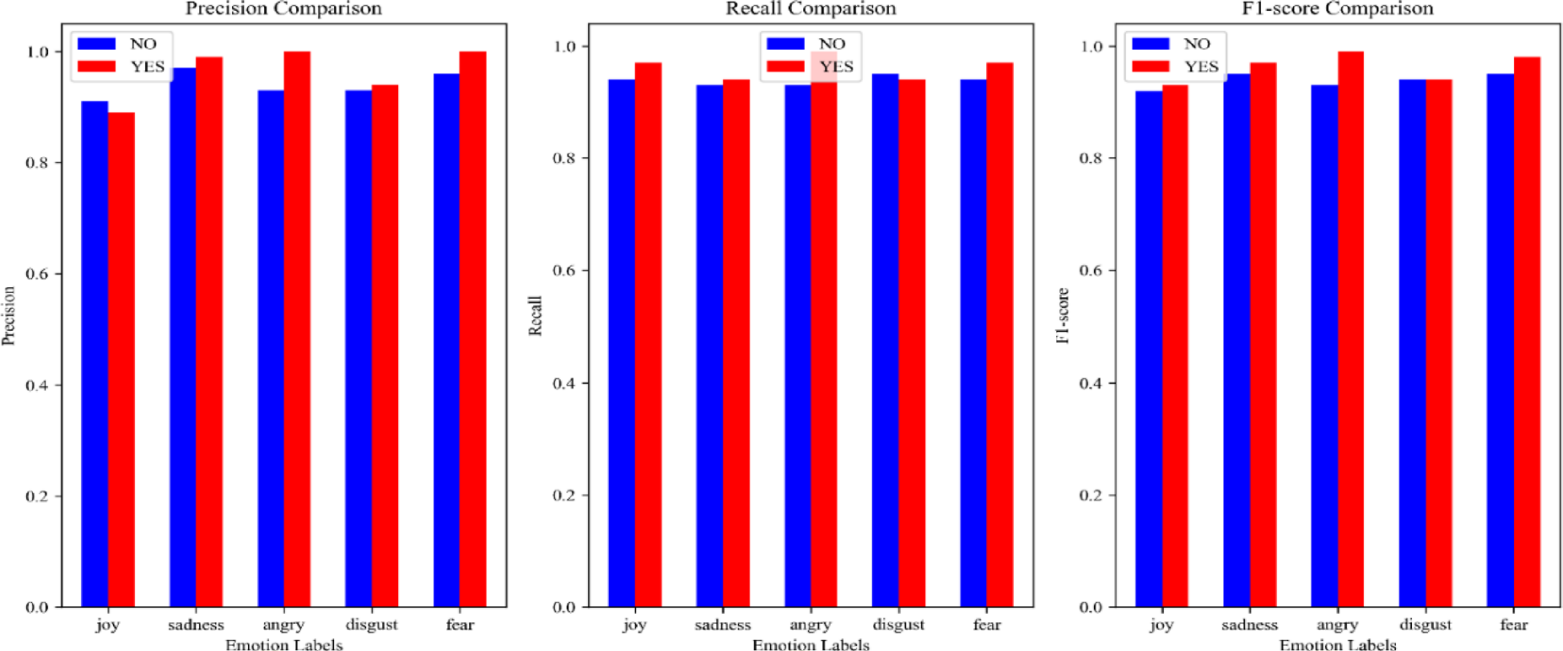
The Precision, Recall, and F1 score of GCN are evaluated, using graph coarsening and without using graph coarsening separately. The blue bar represents the results without using graph coarsening, and the red bar represents the results using graph coarsening.

Figure 4 visualizes the performance of two configurations: with and without graph coarsening. Overall, the values of the metrics with graph coarsening are generally higher than those without graph coarsening. Additionally, Table 2 records the values for accuracy, macro average, and weighted average. Clearly, the experimental results with coarsening are consistently higher than those without coarsening. Therefore, based on the experimental results, it can be concluded that using graph coarsening is a better design than not using it.

**Table 2:** The Accuracy, Macro average, and weighted Macro average of GCN are evaluated, using graph coarsening and without using graph coarsening separately.

Condition	Accuracy^59^	Macro avg^60^	Weighted avg^60^
No coarsening	0.94	0.94	0.94
Coarsening	0.96	0.96	0.96

## Experiments and discussion

The introduction section thoroughly outlines the challenges encountered by previous algorithms, how the technique proposed in this paper addresses these challenges, and the performance of the proposed method. This section will provide a detailed description of three experiments, including their experimental design, the metrics employed, the objectives of each experiment, and a discussion of the results.

### Using Erdős–Rényi graph

This experiment aims to evaluate which of the applied label propagation algorithms yields partitions that are more effectively learned by a GCN^61–63^. Superior learning performance implies that the corresponding community structure is more prominent and meaningful. The algorithms compared include the asynchronous LPA algorithm, the Louvain algorithm, the Fast LPA algorithm, the Greedy Modularity algorithm, and the AME algorithm. In this experiment, the Erdős–Rényi graph^23,24^ model is employed to generate the test graphs used for evaluating the performance of different algorithms.

The evaluation metrics of this experiment are training accuracy^64^, validation accuracy^65^, negative log likelihood loss^66^, and final test accuracy^64^. Loss value and accuracy are important metrics used to evaluate the performance of machine learning models. The loss value measures the quality of the model’s predictions and classifications, with smaller values indicating better performance. Accuracy is a percentage that represents the proportion of correct predictions, with higher values being better. Validation accuracy refers to the model’s prediction accuracy on the validation set. It is used to monitor the training process and to determine whether the model is overfitting. A low validation accuracy may indicate overfitting, while a higher validation accuracy suggests that the model has learned well. In conclusion, validation accuracy is used to monitor whether the model is overfitting; a higher value suggests better generalization. Test accuracy evaluates the model’s performance on unseen data, where a higher value indicates stronger predictive ability. The loss value measures the quality of the model’s predictions, with lower values corresponding to better performance.

The design of this experiment consists of four steps. The first step involves generating a probabilistic graph, which serves as the initial input for the comparison algorithms. In this experiment, the Erdős–Rényi probability model is used to construct the probabilistic graph. Notably, the generated graph models and real-world graph datasets, including the Erdős–Rényi Graph model, the Barabási–Albert Graph model, the Facebook social circles dataset^30^, and the email network dataset^31,32^, do not contain any initial emotional labels. To estimate these labels, the Emotion English DistilRoBERTa-base transformer model^29^ was applied to two real-world text datasets: the Kaggle Emotion Detection dataset^27^ and the CARER dataset^28^.

For each dataset, the textual elements were aligned with the corresponding graph nodes based on their indices. Each text segment was assigned to a specific node, with padding applied as needed to ensure proper alignment. The DistilRoBERTa-base model^29^ was then employed to process the textual content associated with each node, generating a probability distribution over the defined emotion categories. These distributions were subsequently used as the initial emotional label probabilities for the corresponding graph nodes.

To initialize vertex weights in the graph, each text message undergoes preprocessing and classification to generate a probability distribution over emotion labels. First, the input text is padded and aligned according to the index of corresponding vertices in the graph structure. This experiment utilized two real-world datasets, the Kaggle Emotion Detection dataset^27^ and the CARER dataset^28^, to independently initialize node weights. The alignment between graph nodes and textual elements was achieved by matching their indices, with padding applied as necessary to ensure consistency. A Transformer-based model was employed to process the textual elements linked to all graph nodes, producing a probability distribution across predefined emotional categories. These probabilities were used as the initial emotional weights for the corresponding nodes. For example, a sample output may indicate a high likelihood of the message expressing joy (score: 0.977), with much lower probabilities assigned to other emotions such as anger (0.004), disgust (0.0016), and fear (0.0004). These probability values are then used to assign initial weights to the respective vertices in the graph, reflecting the emotional tendency of each message. This process effectively transforms unstructured textual input into structured, weighted graph nodes that are emotion-aware, supporting subsequent analysis tasks.

In the second step, each community detection algorithm is applied to the social network generated in the first step to obtain the corresponding community partitions. A critical part of this step is loading the social network from the first step. Each vertex is associated with a unique probability distribution generated by the Emotion English DistilRoBERTa-base transformer^29^. After loading the social network, five community detection algorithms are applied: the AME algorithm, the asynchronous LPA algorithm, the Louvain algorithm, the Fast LPA algorithm, and the Greedy Modularity algorithm^9^. These community detection algorithms are used to partition the social network, and the resulting partitions are saved for subsequent experimental analysis. In summary, this step executes various community detection algorithms and stores the partition results for the next step, where their performances are compared.

The third step involves evaluating different community detection results as training datasets for a deep learning model, with the goal of identifying which partition yields better learning and validation performance. This step begins by loading the partition result, which records the community index of each vertex in the social network. These community indices serve as labels for the corresponding vertices in the graph, enabling supervised learning. Next, prepare features and graph data by creating an identity matrix as features, using one-hot encoding, remapping node IDs to a contiguous index starting from 0, and converting edges to tensor format. Then, split the dataset into training, validation, and test sets, with a ratio distribution of 60% for training, 20% for validation, and 20% for testing. After completing the previous steps, define the deep learning model. In this experiment, a GCN^8,9,39^ is used for training, and validation accuracy, test accuracy, and negative log-likelihood loss are employed to ensure that the model generalizes well and avoids overfitting. The training function executes one epoch of training by forward propagating the node features through the model, computing the loss value using negative log-likelihood on the training data, and updating the weights via backpropagation using the Adam optimizer. In conclusion, this step performs parameter updates to minimize the classification loss over the training nodes, laying the groundwork for the next step: comparing which community detection algorithm’s partition results are most suitable for effective learning by the GCN model.

In the fourth step, the trained model is evaluated on a test dataset to assess its final accuracy performance, in order to determine which community detection algorithm produces a partitioning that is more effective for learning with the GCN model. The evaluation function performs inference on the entire set of nodes without enabling gradient tracking. It predicts class labels by computing the argmax of the log-softmax outputs, and calculates the accuracy on both the validation and test subsets. This step aims to measure the model’s performance in correctly predicting node labels on unseen validation and test sets. A more effective community detection algorithm results in a lower loss value and higher test and validation accuracy.

**Fig. 5:**
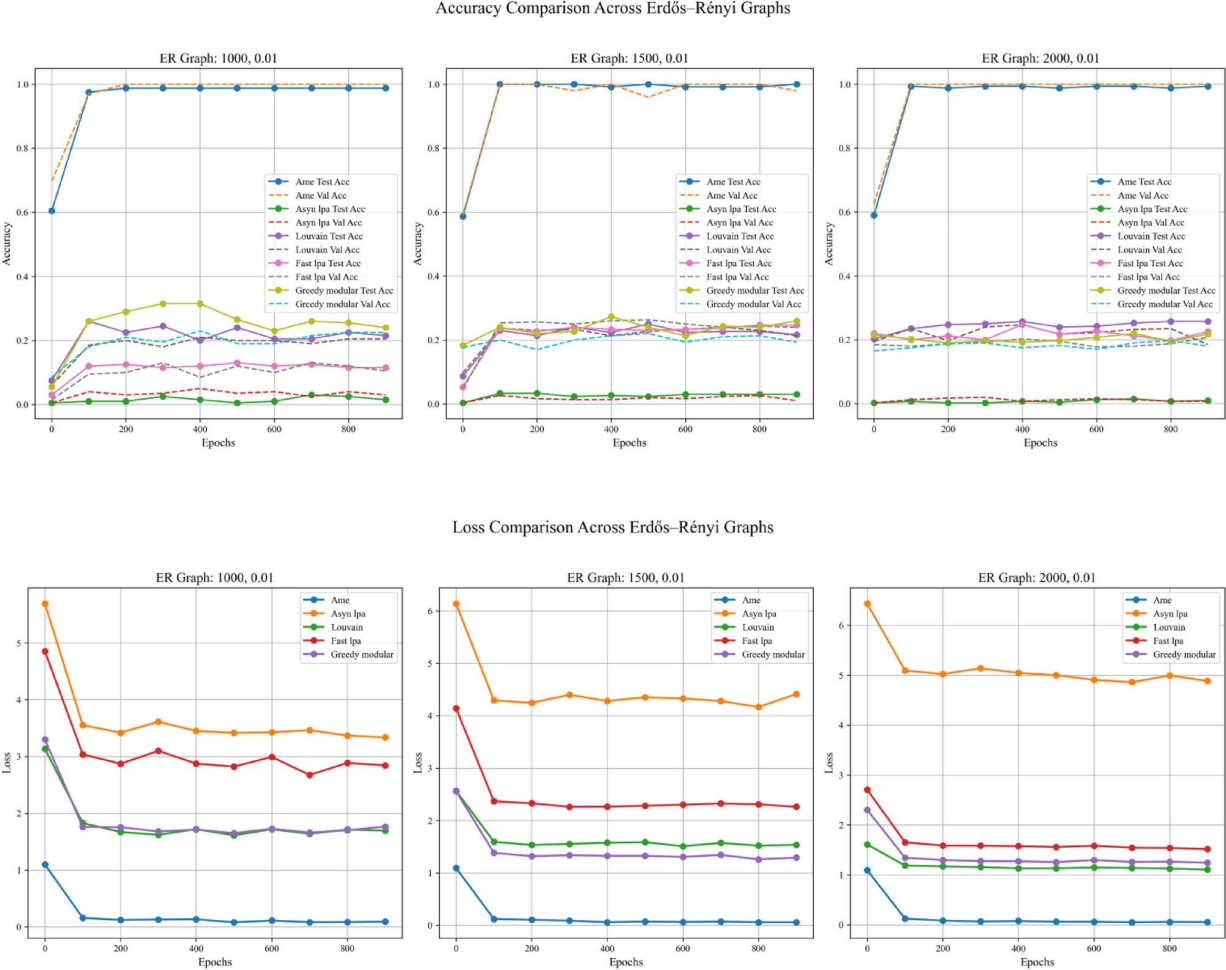
In each plot, the solid lines represent the test accuracy of different methods, while the dashed lines indicate their validation performance. The blue, green, purple, pink, and lime solid lines correspond to the AME algorithm, the asynchronous label propagation algorithm^6^, the Louvain algorithm^7^, the fast label propagation algorithm^8^, and the greedy modular algorithm^9^, respectively. Similarly, the orange, red, brown, gray, and cyan dashed lines represent the validation performance of the same algorithms in the same order. It is evident that the AME algorithm achieved the best overall performance across the three graphs.

**Fig. 6:**
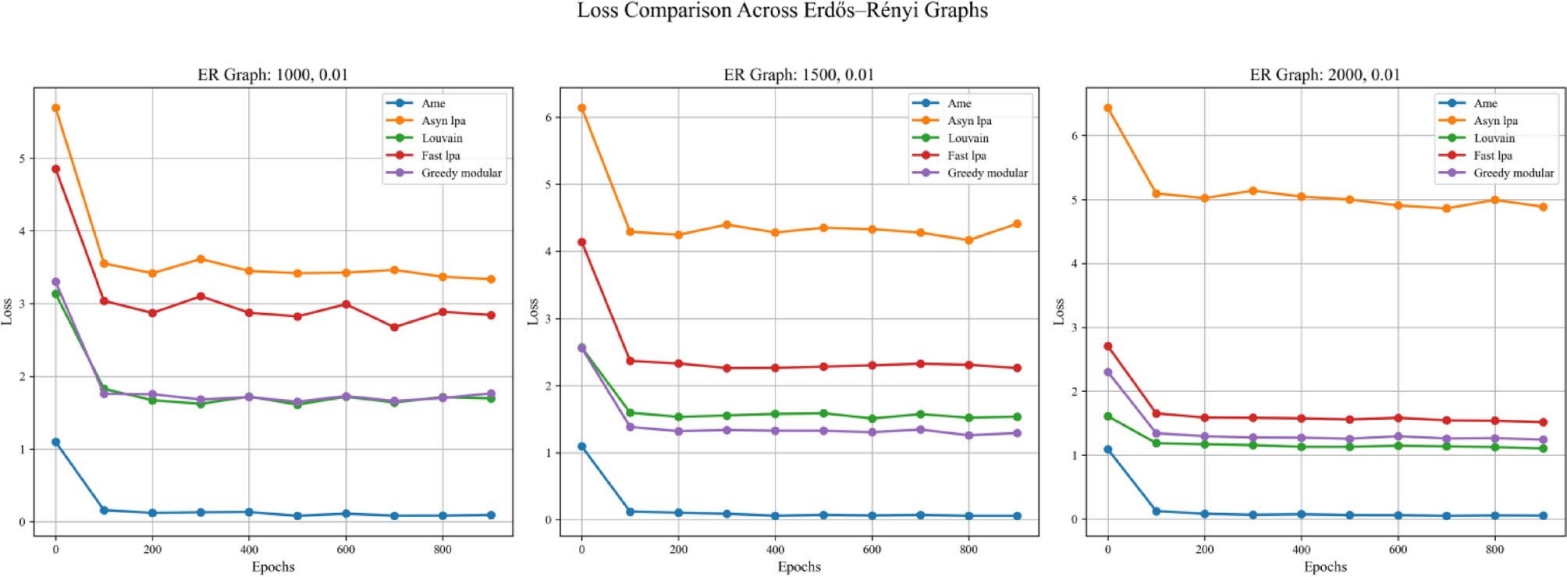
In each plot, the dashed lines represent the performance on the validation set. The blue, orange, green, red, and purple solid lines correspond to the AME algorithm, the asynchronous label propagation algorithm^6^, the Louvain algorithm^7^, the fast label propagation algorithm^8^, and the greedy modular algorithm^9^, respectively. Notably, the AME algorithm achieved the lowest loss value across all three graphs.

**Fig. 7:**
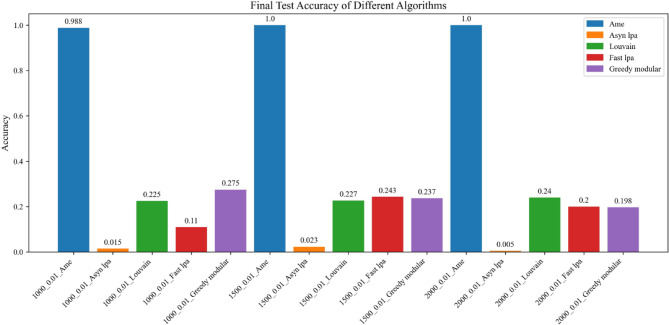
The test accuracy is measured using the trained model. The blue, orange, green, red, and purple bars correspond to the AME algorithm, the asynchronous label propagation algorithm^6^, the Louvain algorithm^7^, the fast label propagation algorithm^8^, and the greedy modular algorithm^9^, respectively. Notably, the AME algorithm achieved the highest test accuracy across all three graphs.

**Fig. 8:**
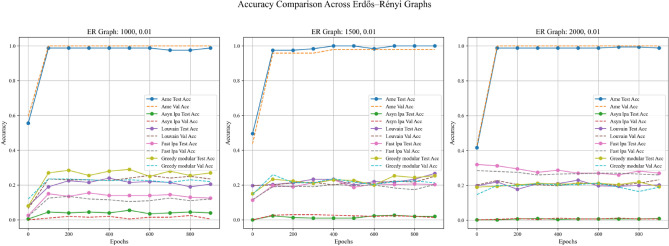
In each plot, the solid lines represent the test accuracy of different methods, while the dashed lines indicate their performance on the validation set. The blue, green, purple, pink, and lime solid lines correspond to the AME algorithm, the asynchronous label propagation^6^ algorithm, the Louvain algorithm^7^, the fast label propagation algorithm^8^, and the greedy modular algorithm^9^, respectively. Similarly, the orange, red, brown, gray, and cyan dashed lines represent the validation performance of the same algorithms in the same order. The AME algorithm consistently outperformed the others across all three graphs.

**Fig. 9:**
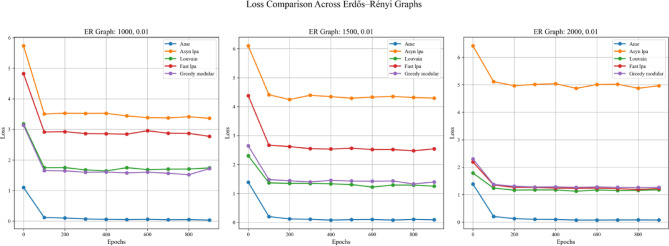
In each plot, the dashed lines indicate their performance on the validation set. The blue, orange, green, red, and purple solid lines correspond to the AME algorithm, the asynchronous label propagation^6^ algorithm, the Louvain algorithm^7^, the fast label propagation algorithm^8^, and the greedy modular algorithm^9^. Notably, the AME algorithm achieved the lowest loss value across three graphs.

**Fig. 10:**
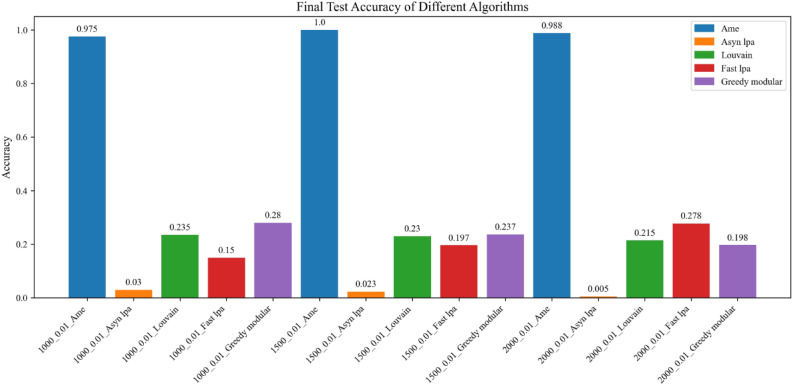
The test accuracy is evaluated using the trained model. The blue, orange, green, red, and purple bars correspond to the AME algorithm, the asynchronous label propagation algorithm^6^, the Louvain algorithm^7^, the fast label propagation algorithm^8^, and the greedy modular algorithm^9^, respectively. Notably, the AME algorithm achieved the highest test accuracy value across three graphs.

In addition to the four aforementioned steps, this experiment also examines the impact of employing different real-world datasets in Step 1, with the aim of more thoroughly evaluating the generalization capabilities of the algorithms. Specifically, the experiment was conducted twice, differing only in the real-world text dataset used for initializing node weights in Step 1. The two datasets employed were the Kaggle Emotion Detection dataset (Gupta, n.d.)^27^ and the CARER dataset^28^. Figures 5, 6, and 7 present the results obtained using the Kaggle Emotion Detection dataset ^27^, while Figs. 8, 9, and 10 show the results based on the CARER dataset^28^. This approach enables a more comprehensive comparison of each algorithm’s generalization performance. A well-performing algorithm is expected to exhibit higher validation and test accuracy, as well as lower loss values.

Based on the results, the AME algorithm demonstrates the best performance in this experiment. Figures 5, 6, 8, and 9 illustrate the training performance of different Erdős–Rényi graphs with respect to both accuracy and loss values. Figures 5 and 6 were initialized using the Kaggle Emotion Detection dataset^27^, while Figs. 8 and 9 were initialized using the CARER dataset^28^ to provide attribute information in the first step of the process. Across all six Erdős–Rényi graph configurations, the AME algorithm consistently achieves the highest test and validation accuracy, as well as the lowest loss values during training. These results clearly indicate the superior performance of the AME algorithm in terms of both learning and generalization throughout the training process. Figures 7 and 10 visualize the accuracy of the trained model on the test dataset. Figure 7 is initialized with the Kaggle Emotion Detection dataset^27^, while Fig. 10 is initialized using the CARER dataset ^28^. Consistently, the AME algorithm achieves the highest accuracy across all six Erdős–Rényi graph configurations. These results further substantiate that the AME algorithm demonstrates superior effectiveness compared to the other methods.

### Using Barabási–Albert graph

This experiment aims to assess the effectiveness of different label propagation algorithms within the framework of a GCN. An effective algorithm is expected to yield higher accuracy and lower loss value. The experimental setup is based on the Barabási–Albert Graph^25,26^, which incorporates a preferential attachment mechanism. Experiment 2 employs the Barabási–Albert graph as a generative model to facilitate a more comprehensive evaluation of label propagation algorithms. Validation accuracy, test accuracy, and loss value serve as the evaluation metrics in this experiment. Under optimal conditions, the label propagation algorithm achieved the highest validation accuracy and test accuracy, with the lowest loss value. The overall design follows that of Experiment 1, with the key difference being the replacement of the generated graph model with the Barabási–Albert graph.

**Fig. 11:**
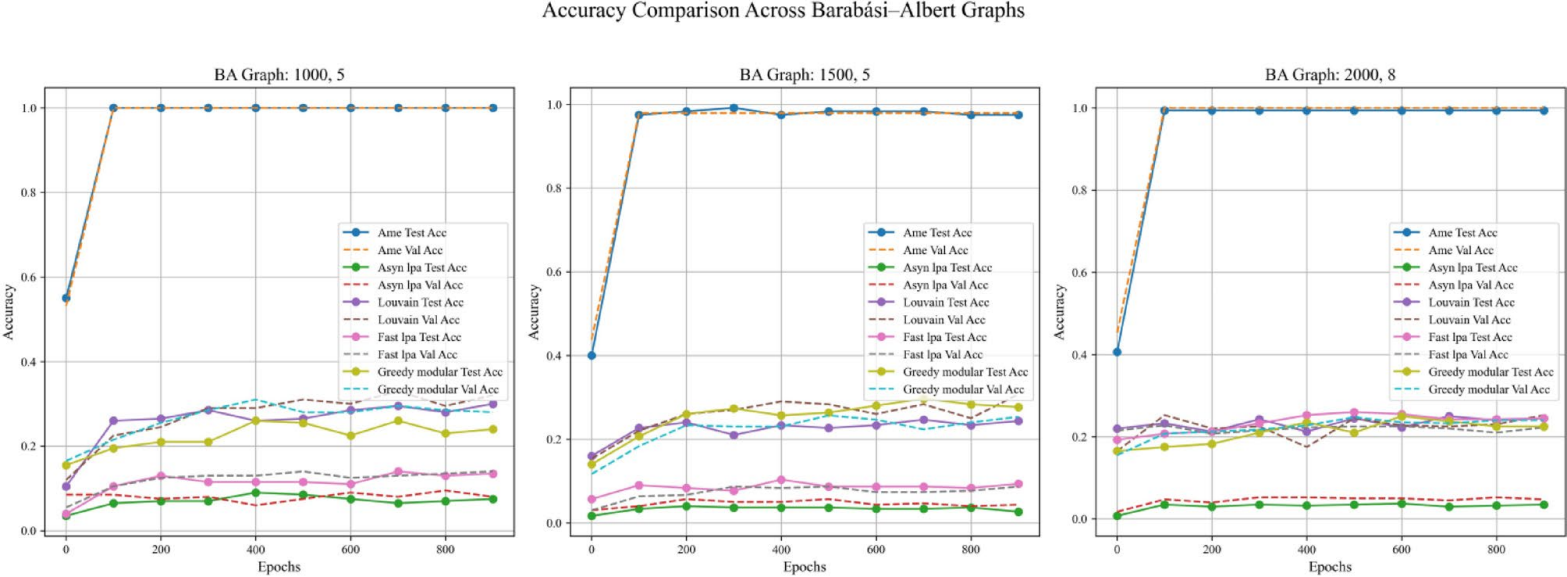
In each graph, the solid lines depict the test accuracy for various methods, while the dashed lines show their performance on the validation set. The solid blue, green, purple, pink, and lime lines represent the AME algorithm, the asynchronous label propagation^6^ algorithm, the Louvain algorithm^7^, the fast label propagation algorithm^8^, and the greedy modular algorithm^9^, respectively. Likewise, the dashed orange, red, brown, gray, and cyan lines illustrate the validation performance of these same algorithms in the same order. It is clear that the AME algorithm outperformed the others across all three graphs.

**Fig. 12:**
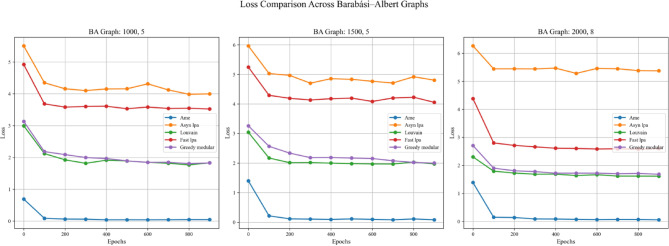
In each plot, the dashed lines represent the performance on the validation set. The solid blue, orange, green, red, and purple lines correspond to the AME algorithm, the asynchronous label propagation^6^ algorithm, the Louvain algorithm^7^, the fast label propagation algorithm^8^, and the greedy modular algorithm^9^. Notably, the AME algorithm achieved the lowest loss value across all three graphs.

**Fig. 13:**
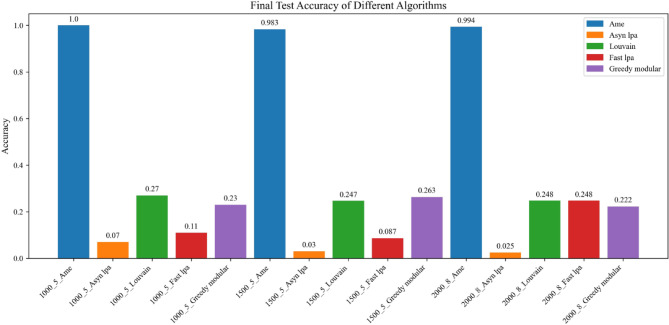
Test accuracy is evaluated after training the model. The blue, orange, green, red, and purple bars represent the AME algorithm, the asynchronous label propagation^6^ algorithm, the Louvain algorithm^7^, the fast label propagation algorithm ^8^, and the greedy modular algorithm^9^, respectively. Notably, the AME algorithm achieved the highest test accuracy across all three graphs.

**Fig. 14:**
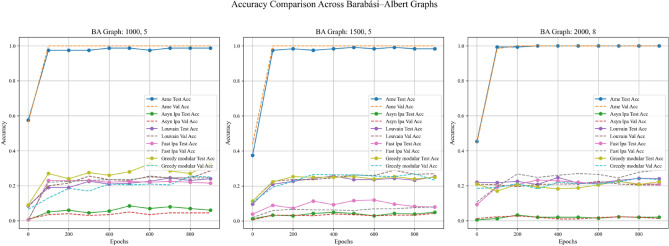
In each graph, the solid lines depict the test accuracy for various methods, while the dashed lines show their performance on the validation set. The blue, green, purple, pink, and lime solid lines represent the AME algorithm, the asynchronous label propagation^6^ algorithm, the Louvain algorithm^7^, the fast label propagation algorithm^8^, and the greedy modular algorithm^9^, respectively. Similarly, the orange, red, brown, gray, and cyan dashed lines illustrate the validation performance of these same algorithms in the same order. It is evident that the AME algorithm consistently outperformed the other methods across all three graphs.

**Fig. 15:**
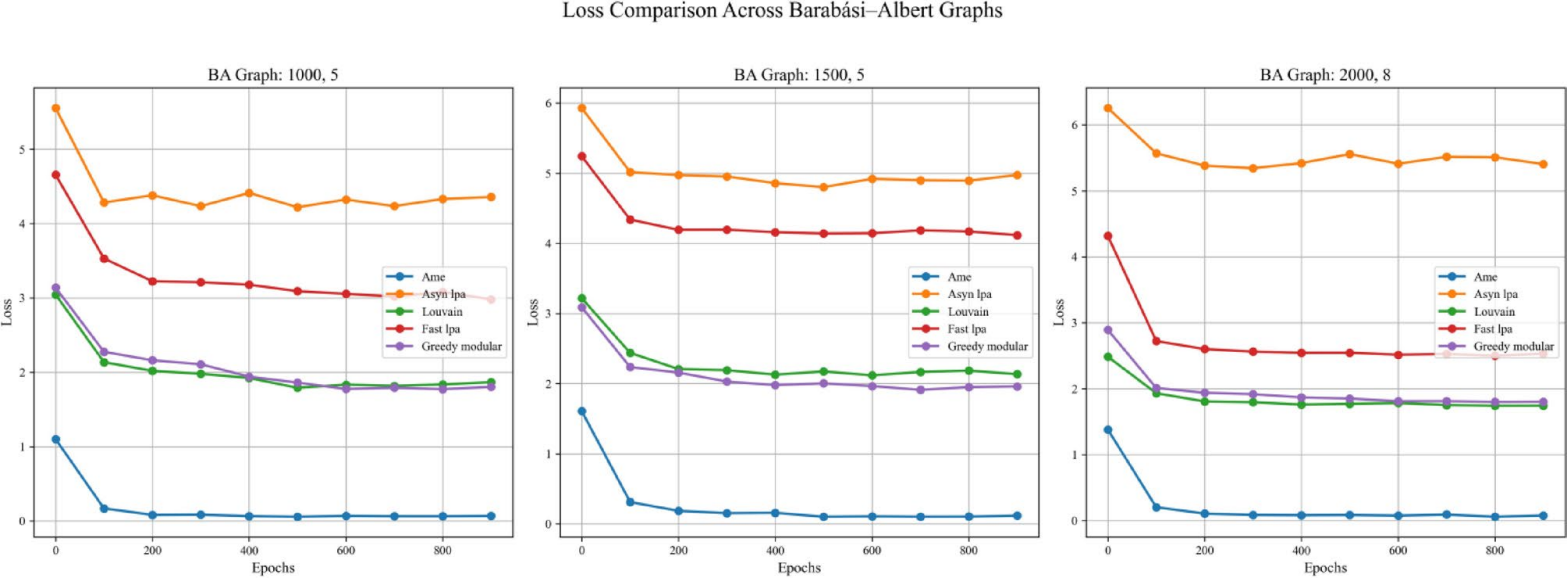
In each plot, the dashed lines represent the performance on the validation set. The solid blue, orange, green, red, and purple lines correspond to the AME algorithm, the asynchronous label propagation^6^ algorithm, the Louvain algorithm^7^, the fast label propagation algorithm^8^, and the greedy modular algorithm^9^. Notably, the AME algorithm achieved the lowest loss value across all three graphs.

**Fig. 16:**
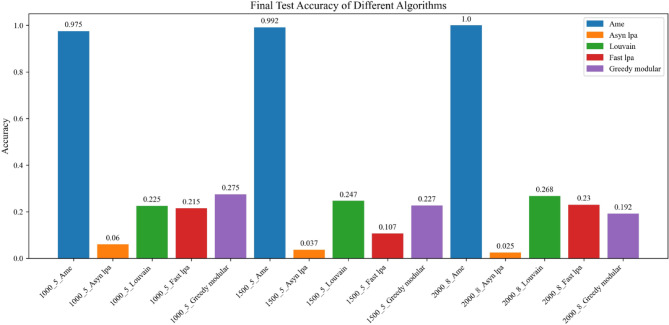
The test accuracy is measured after training the model. The blue, orange, green, red, and purple bars represent the AME algorithm, the asynchronous label propagation ^6^ algorithm, the Louvain algorithm^7^, the fast label propagation algorithm^8^, and the greedy modular algorithm^9^, respectively. Notably, the AME algorithm achieved the highest test accuracy across all three graphs.

In addition, this experiment employed two real-world datasets to initialize node weights separately, in order to validate the generalization ability of different community detection algorithms. A critical step in the process involves mapping textual elements from the real-world datasets to graph nodes based on index alignment. When a node shares the same index as a dataset element, the corresponding text is assigned to that vertex. Next, the Emotion English DistilRoBERTa-base fine-tuned transformer model^29^ is used to encode the emotional probability of each textual element. This process loops through the elements associated with each vertex until all vertices in the graph are processed. These probabilities are used as weights during training. In summary, this method enhances the overall robustness and completeness of the experiment. Figures 11, 12, and 13 are based on the Kaggle Emotion Detection dataset (Gupta, n.d.)^27^, while Figs. 14, 15, and 16 are based on the CARER dataset^28^. This design supports a more rigorous assessment of the generalization ability of different community detection algorithms.

Based on the experimental results, the AME algorithm demonstrates the best overall performance. Figures 11, 12, and 13 are based on the Kaggle Emotion Detection dataset^27^, while Figs. 14, 15, and 16 are initialized using the CARER dataset^28^. Figures 11, 12, 14, and 15 illustrate the training performance of the models on different Barabási–Albert graphs. The AME algorithm achieves the highest test and validation accuracy, along with the lowest loss value on each Barabási–Albert graph. Meanwhile, Figs. 13 and 16 visualize the accuracy of the trained models on the test dataset. The AME algorithm achieves the highest test accuracy of all test datasets. These results clearly demonstrate the effectiveness of the AME algorithm.

### Using real-world benchmarks

The design of this experiment aims to compare the performance of various algorithms across different real-world datasets. In Experiments 1 and 2, the graphs were generated based on mathematical models, namely the Erdős–Rényi model and the Barabási–Albert model, respectively. Each of these generative models possesses unique structural characteristics, which can influence algorithm performance in varying ways. Therefore, the objective of this experiment is to evaluate and compare the performance of different algorithms on real-world datasets. The evaluation metrics used in this experiment include validation accuracy, test accuracy, and negative log-likelihood loss. The optimal outcome is high validation accuracy and test accuracy, with a low loss value. In line with this objective, the experimental design is consistent with that of Experiment 1. The synthetic datasets were replaced with two real-world graphs^27–29^, namely the Social circles from Facebook^30^ and the E-mail network^31,32^.

**Fig. 17:**
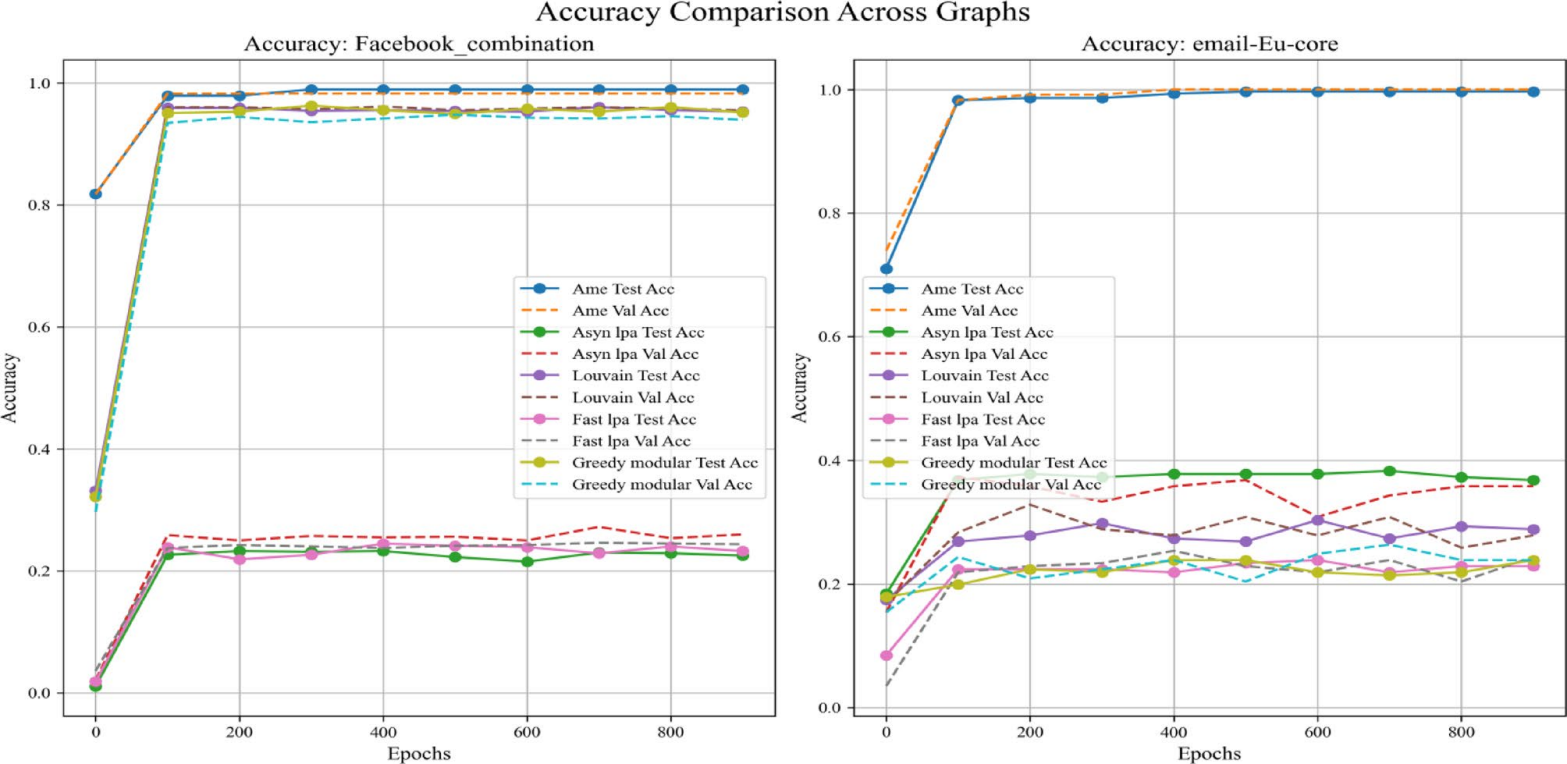
In every plot, the solid lines depict the test accuracy of various methods, while the dashed lines show their performance on the validation set. The solid blue, green, purple, pink, and lime lines represent the AME algorithm, the asynchronous label propagation algorithm^6^, the Louvain algorithm^7^, the fast label propagation algorithm^8^, and the greedy modularity algorithm^9^, respectively. Likewise, the dashed orange, red, brown, gray, and cyan lines reflect the validation performance of the same algorithms in the same sequence. Clearly, the AME algorithm consistently outperformed the others across all three graphs.

**Fig. 18:**
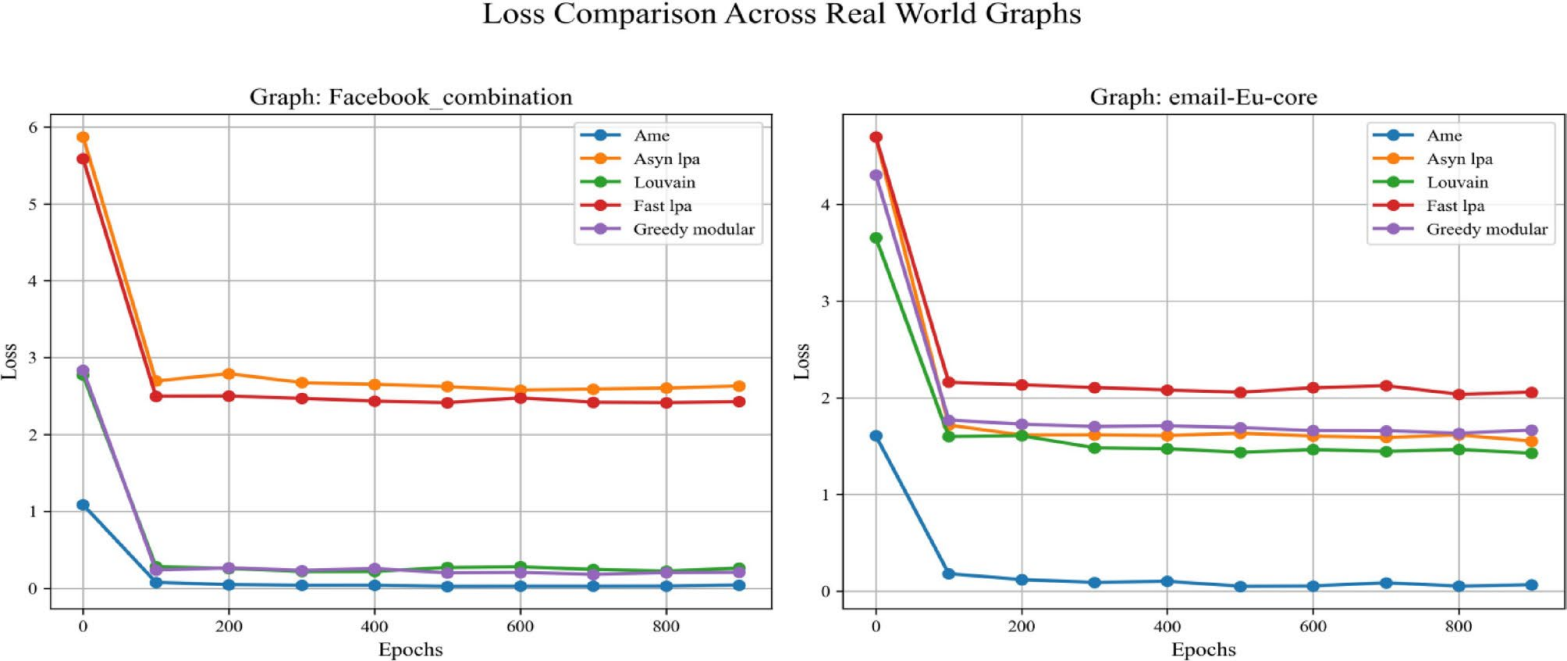
In each plot, the dashed lines represent the performance on the validation set. The solid blue, orange, green, red, and purple lines correspond to the AME algorithm, the asynchronous label propagation algorithm ^6^, the Louvain algorithm^7^, the fast label propagation algorithm^8^, and the greedy modularity algorithm ^9^, respectively. Notably, the AME algorithm achieved the lowest loss value across all three graphs.

**Fig. 19:**
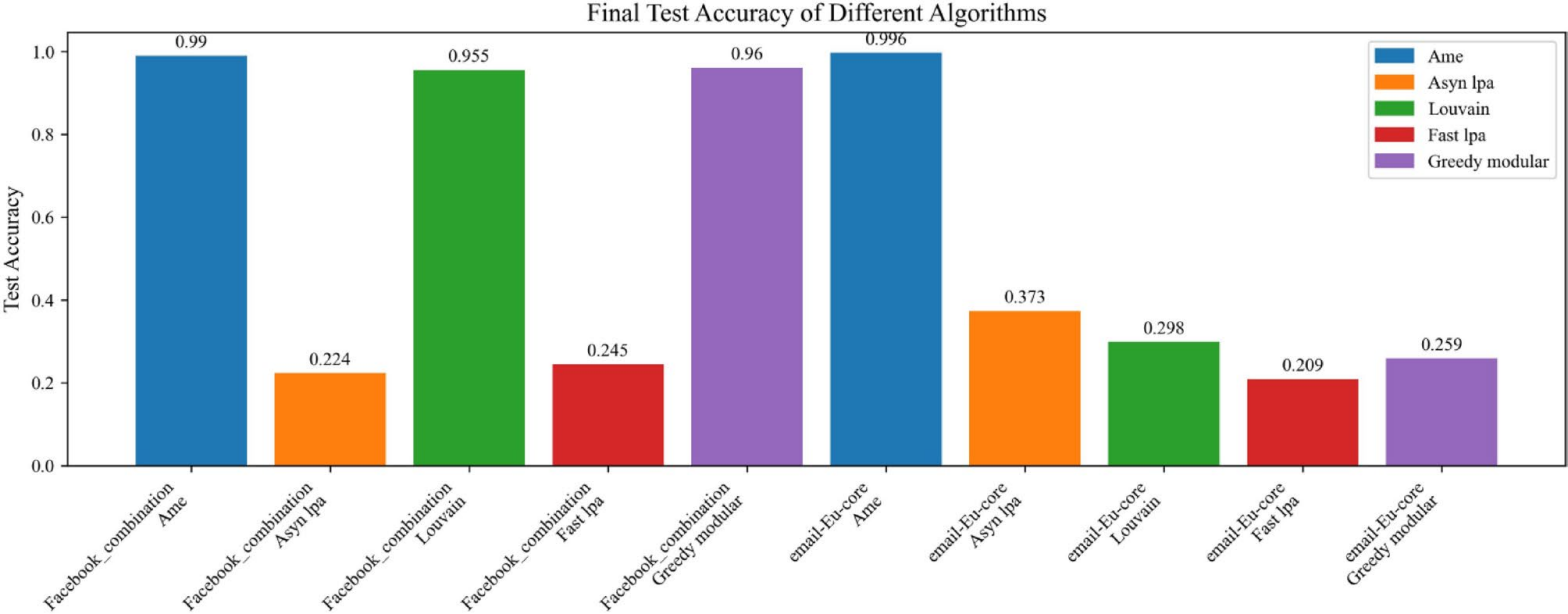
The test accuracy is assessed by training the model. The Blue bar, orange bar, green bar, red bar, and purple bar represent the AME algorithm, the asynchronous label propagation^6^ algorithm, the Louvain algorithm^7^, the fast label propagation algorithm^8^, and the greedy modular algorithm^9^, respectively. Notably, the AME algorithm achieved the highest test accuracy across all three graphs.

**Fig. 20:**
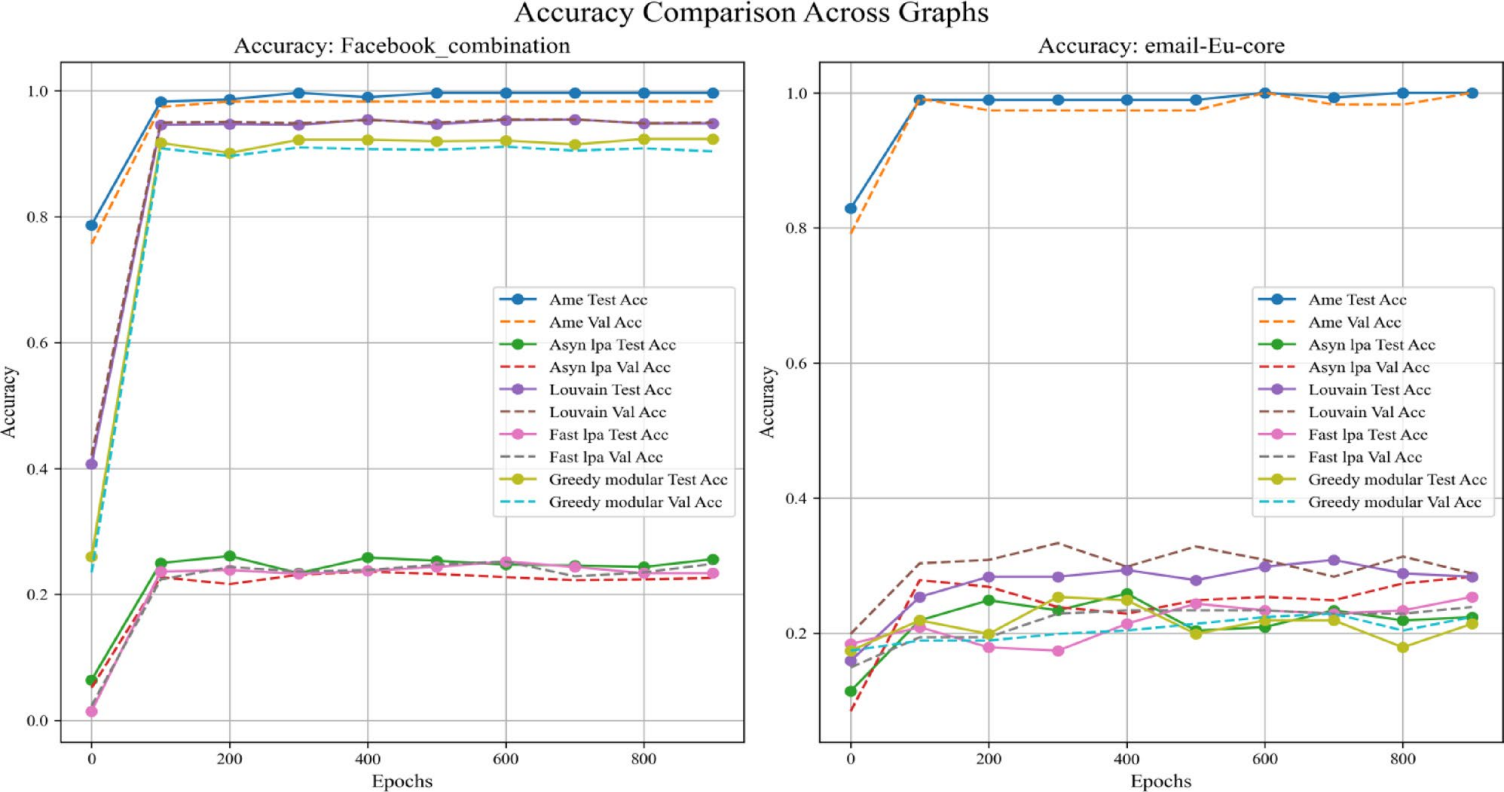
In every plot, the solid lines depict the test accuracy of various methods, while the dashed lines show their performance on the validation set. The blue, green, purple, pink, and lime solid lines represent the AME algorithm, the asynchronous label propagation algorithm^6^, the Louvain algorithm^7^, the fast label propagation algorithm^8^, and the greedy modularity algorithm^9^, respectively. Likewise, the orange, red, brown, gray, and cyan dashed lines reflect the validation performance of the same algorithms in the same order. Clearly, the AME algorithm outperformed the others in all three graphs.

**Fig. 21:**
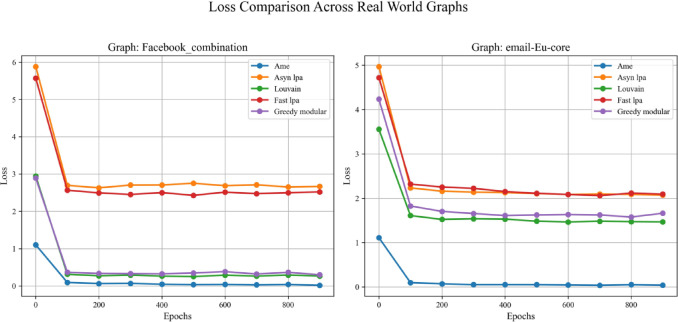
In each plot, the dashed lines represent the performance on the validation set. The blue, orange, green, red, and purple solid lines correspond to the AME algorithm, the asynchronous label propagation algorithm^6^, the Louvain algorithm^7^, the fast label propagation algorithm^8^, and the greedy modularity algorithm ^9^, respectively. Notably, the AME algorithm attained the lowest loss value across all three graphs.

**Fig. 22:**
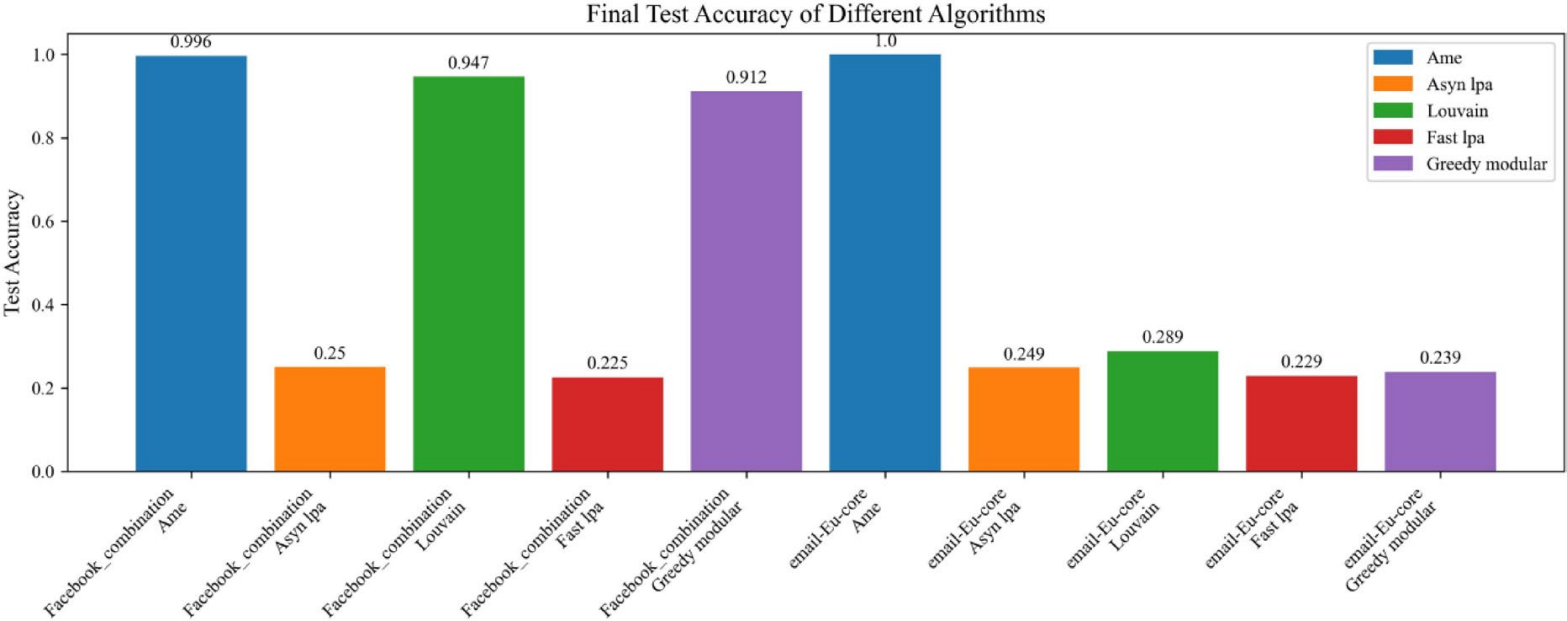
The test accuracy is assessed by training the model. The blue, orange, green, red, and purple bars represent the AME algorithm, the asynchronous label propagation algorithm^6^, the Louvain algorithm^7^, the fast label propagation algorithm^8^, and the greedy modularity algorithm^9^, respectively. Notably, the AME algorithm achieved the highest test accuracy across all three graphs.

Moreover, this experiment separately employed different real-world datasets to evaluate the generalization ability of various community detection algorithms, including the Assimilation Modified Emotional algorithm, the asynchronous LPA algorithm, the Louvain algorithm, the Fast LPA algorithm, and the Greedy Modular algorithm^9^. Figures 17, 18, and 19 utilize the Kaggle Emotion Detection dataset^27^, while Figs. 20, 21, and 22 use the CARER dataset^28^. This design facilitates a more thorough assessment of various algorithms’ performance, enabling the identification of the most optimal algorithm.

Figures 17, 18, and 19 use the Kaggle Emotion Detection dataset^27^, while Figs. 20, 21, and 22 use the CARER dataset^28^. According to the experiment results, Figs. 17, 18, 20, and 21 illustrate the training-validation accuracy, test accuracy, and loss values during the GCN training process. Community detection was performed using the AME algorithm, the asynchronous label propagation algorithm ^6^, and the Louvain algorithm^7^. The AME algorithm achieved the best scores across all three metrics. Meanwhile, Figs. 19 and 22 visualize the final test accuracy obtained by each GCN. It is evident that, regardless of whether the graph is the Facebook graph or the Email-EU-Core graph, the AME algorithm consistently achieved the best results. Therefore, it can be concluded that the AME algorithm demonstrated the most outstanding performance on the real-world datasets used in this experiment.

## Conclusion

This paper proposes a novel label propagation algorithm, the AME algorithm, which enhances emotional feature analysis in the context of social network analysis. Unlike traditional label propagation algorithms (LPA), which fail to capture emotional features in their label representations, the AME algorithm effectively incorporates the assimilation effect^15–18^ into the label propagation process. Furthermore, the new labels and graph updates generated by the AME algorithm demonstrate superior performance in AI training, showcasing a robust update structure that enhances AI learning. The AME algorithm holds great potential for integration with AI techniques.

The primary advantage of the AME algorithm is its ability to address two key limitations: reliance on local structural information and the absence of label encoding. Traditional label propagation algorithms suffer from these limitations. The AME algorithm employs multiple probability distributions, Markov chains^19–21^, and graph coarsening to address them, and it achieves optimized results across each experiment.

Experiments conducted using Erdős–Rényi Graph^23,24^, Barabási–Albert graph^25,26^, and Real-World benchmarks^27–29^ demonstrated the effectiveness of the AME algorithm. In each experiment graph, the AME algorithm achieved the best performance. These results not only reflected the robustness of the AME algorithm but also demonstrated its generalized ability. Validation experiments on the encoding design and graph coarsening design confirmed the convergence of core components; samples applying the core design achieved optimized performance.

In summary, the AME algorithm outperforms traditional label propagation algorithms in social network analysis, making it a promising tool with strong potential for AI-enhanced emotional feature extraction and network-based learning tasks.

## Data Availability

The datasets and fine-tuning AI deep learning transformer model analysed during the current study are available in the NetworkX repository, SNAP, Kaggle and Hugging Face. The link is https://networkx.org/documentation/stable/reference/generators.html. And https://snap.stanford.edu/data. Erdős–Rényi Graph is located at https://networkx.org/documentation/stable/reference/generated/networkx.generators.random_graphs.erdos_renyi_graph.html. Barabási–Albert Graph is located at https://networkx.org/documentation/stable/reference/generated/networkx.generators.random_graphs.barabasi_albert_graph.html. Social circles from the Facebook graph are located at https://snap.stanford.edu/data/ego-Facebook.html. The email network graph is located at https://snap.stanford.edu/data/email-Eu-core.html. The CARER dataset is located at https://www.kaggle.com/datasets/parulpandey/emotion-dataset. The Kaggle Emotion Detection dataset is located at https://www.kaggle.com/datasets/pashupatigupta/emotion-detection-from-text. The Fine-tuning Transformer model, named emotion-english-distilroberta-base, is located at https://huggingface.co/j-hartmann/emotion-english-distilroberta-base.
